# Rhodamine 6G conjugated to gold nanoparticles as labels for both SERS and fluorescence studies on live endothelial cells

**DOI:** 10.1007/s00604-014-1307-5

**Published:** 2014-06-19

**Authors:** Aleksandra Jaworska, Tomasz Wojcik, Kamilla Malek, Urszula Kwolek, Mariusz Kepczynski, Abu A. Ansary, Stefan Chlopicki, Malgorzata Baranska

**Affiliations:** 1 Jagiellonian Centre for Experimental Therapeutics (JCET), Jagiellonian University, Bobrzynskiego 14, 30-348 Krakow, Poland; 2 Faculty of Chemistry, Jagiellonian University, Ingardena 3, 30-060 Krakow, Poland; 3 Department of Experimental Pharmacology, Jagiellonian University Medical College, Grzegorzecka 16, 31-531 Krakow, Poland

**Keywords:** Endothelium, SERS imaging, 2D and 3D fluorescence, Rhodamine 6G, Cellular uptake

## Abstract

Fluorescence and surface-enhanced Raman scattering (SERS) spectroscopy were employed to investigate the cellular uptake of rhodamine 6G (R6G) alone and of R6G loaded with gold nanoparticles (AuNPs) by endothelial cells. R6G plays the role of a Raman reporter in SERS but also displays strong fluorescence. The presence of bare R6G molecules and R6G-AuNPs in the cytoplasm of the cells is detected via the 2D fluorescence of the dye after a 0.5 h of the incubation with R6G and R6G-AuNPs, and then the concentration of the dye increases within 4 h of exposure. The examination of the cellular uptake of the R6G and R6G-AuNPs species at different temperatures suggests that the internalization of the R6G-AuNPs into endothelial cells occurs mainly via endocytosis. 3D fluorescence imaging of R6G inside cells reveals inhomogeneous distribution of the dye in the cytoplasm. The SERS signal of the Raman reporter inside the cell disappears after 2 h of incubation with R6G-AuNPs and then amino acid residues, purines and pyrimidines become SERS-active via their interactions with the gold. The results highlight the significance of using multiple techniques to cover a spectrum of issues in the application of SERS nanosensors for probing an intracellular environment under comparable and standardized conditions.

**Electronic supplementary material:**

The online version of this article (doi:10.1007/s00604-014-1307-5) contains supplementary material, which is available to authorized users.

**Figure:**
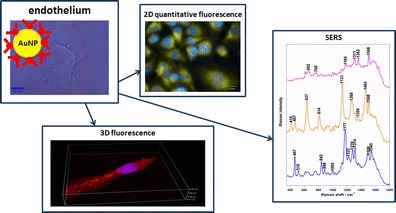
Cellular uptake of bare rhodamine 6G and rhodamine 6G adsorbed onto AuNPs were studied on endothelial cells using fluorescence and surface-enhanced Raman spectroscopy. The internalization of R6G-AuNPs occurs via endocytosis and diffusion resulting in uneven distribution in the cytoplasm.

## Introduction

Raman spectroscopy is a method that enables vibrational modes of individual bonds to be probed optically and has been widely employed in various biomedical experiments [1, 2]. While the Raman scattering is a weak effect challenging to measure spectra from an ultra-low concentration of a sample, for molecules adsorbed on roughened metal surfaces of silver and gold it can be enhanced by up to 14 orders of magnitude by means of surface-enhanced Raman spectroscopy (SERS). This even includes single molecule detection [3, 4]. As a result, Raman cross-sections of molecules interacting with SERS-active substrates can reach values comparable to those observed for the fluorescence phenomenon [3]. SERS has been dynamically developing, especially in the field of bioanalysis, due to its ultrasensitive detection limits [5, 6]. Moreover, informative SERS signals from the intracellular microenvironment can be collected within seconds in contrast to normal Raman scattering [7]. Gold-supported in vitro SERS in living cells provides a tool for sensitive and structurally-selective detection of native chemicals such as DNA, proteins, lipids etc., and for monitoring their intracellular distributions. However, the distribution of bare metal nanoparticles in the cells cannot be controlled [7–9]. Another application of SERS for cellular studies is based on nanoprobes conjugated to Raman reporters [5, 8–11]. The most commonly used reporters are organic dyes, e.g. malachite green, rhodamine B or crystal violet [5–9].

On the other hand, nowadays confocal laser-scanning microscopy (CLSM) is a widely used technique for cellular imaging and biomedical diagnostics [12, 13]. Fluorescence-CLSM is the most appropriate method of imaging cells and subcellular structures, both in vitro and in vivo, once a cell shows intrinsic fluorescence or is labelled with a fluorescent probe [14]. In comparison to the conventional fluorescence microscopy, CLSM reduces the effect of light scattering enabling the observation of thick and light-scattering objects with a good quality [13, 15]. Depending on the numerical aperture of the objective, the excitation wavelength, and the medium refraction index, the spatial resolution in the lateral and axial directions can be as much as 250 nm and >600 nm, respectively [13–15]. Furthermore, this microscopic technique allows the reconstruction of the original three-dimensional structure of the objects in the specimen [13, 16]. As a consequence, CLSM in fluorescence mode provides information on distances between specific structures in the cells and enables imaging of cellular compartments [16] and monitoring physiological parameters and cellular metabolism [12]. Despite the obvious advantages, CLSM has several limitations such as photodamage and photobleaching of the sample [12, 14, 15].

In this work both SERS and fluorescence microscopy are employed to study intracellular environment of endothelial cells. The endothelium is a monolayer of cells lining of all blood vessels (arteries, veins and capillaries) and the lymphatic system, and thus this is in a direct contact with blood/lymph and circulating in blood cells [17]. Currently, this type of the cells is recognized to be a predominant player in the control of blood fluidity, platelet aggregation, thrombosis, vascular tone. Endothelium regulates also immune response, inflammation and angiogenesis [18]. Therefore, endothelial dysfunction, a term that encompasses multiple potential defects of the endothelial cells, can tip the balance toward thrombosis and in consequence this contributes to various pathological states such as atherothrombosis, arterial thrombosis (stroke, visceral and peripheral artery occlusive diseases) and thrombotic microangiopathies [17]. Taking into consideration the importance of endothelium in the regulation of cardiovascular system, novel tools seem mandatory to foster studies on biochemistry and function of endothelial cells.

However, a major challenge of cellular studies in terms of SERS is the delivery of target nanoparticles in sufficient quantities to acquire a spectral signal allowing for simultaneous spatial distribution of the nanoparticle (through a reporter signal) and chemical information of the cellular environment (via the enhancement of Raman signal of biomolecules on a metallic support). Therefore, the SERS- and fluorescence-based studies on the uptake of rhodamine 6G conjugated to gold nanoparticles (R6G-AuNPs) by live endothelial cells have been undertaken in our laboratory. Rhodamine 6G is a fluorophore of a high cross-section for Raman scattering used by us before in cellular studies of macrophages and endothelial cells [8, 9]. For in vitro culture we chose EA.hy926 cells, a line derived from human umbilical vein endothelial cells. Our preliminary investigations have shown variation in SERS information on biocomponents in this cell line along with elongation of incubation time [8]. Thus, we continue our SERS studies with a support of fluorescence microscopy since rhodamine 6G also exhibits a high cross-section for the fluorescence phenomenon. This allows us to follow cellular uptake of R6G-AuNPs and distribution of the Raman reporter inside the cell. Finally, we demonstrate a potential application of this label for probing live endothelial cells.

## Materials and methods

### Materials

Rhodamine 6G (R6G), gold (III) chloride trihydrate, sodium citrate tribasic dihydrate and paraformaldehyde were purchased from Sigma (www.sigmaaldrich.com) and were of analytical grade.

An aqueous stock solution of rhodamine 6G at the concentration of 10^−3^ M was prepared by diluting an appropriate amount of the solid in the 4-fold distilled water. Gold nanoparticles (AuNPs) were prepared according to the Frens procedure [19]. In this synthesis Au ions are reduced by citrate ions. R6G-AuNPs solution was prepared by mixing 10 μL of the 1 × 10^−3^ M solution of R6G with 1 mL of the gold colloid.

### Cells and protocol of SERS and fluorescence experiment

Experiments were conducted for EA.hy926 cells, a line derived from human umbilical vein endothelial cells fused with the A549 line [20]. Cells were grown in 6-well plates in DMEM supplemented with 10 % fetal bovine serum, 2 mM L-glutamine, penicillin, streptomycin, and 2 % HAT. After 24 h passaging a medium was replaced by a medium containing solution of R6G-AuNPs. After 5 min, medium was mixed with the R6G-AuNPs solution (10:1 v/v). The final concentration of R6G and AuNPs in medium was 1 × 10^−6^ and 1 × 10^−11^ M, respectively. Cells were incubated in the medium containing R6G-AuNPs for 0.5, 1, 2, 4 and 16 h. Two control experiments were carried out to exclude autofluorescence of cells and to exclude the contribution of normal Raman signal of the dye non-conjugated to AuNPs. Cells were incubated in a pure medium and a medium containing a pure R6G solution (c = 1 × 10^−6^ M), respectively. Then, the medium was washed out three times with DPBS (Dulbeccos’s phosphate-buffered saline) and cells were kept in a pure medium in an incubator until SERS and 2D fluorescence measurements were performed. For 3D fluorescence measurements, cells were fixed with 4 % paraformaldehyde for 6 min and store in a fridge until measurements. For the all fluorescence measurements, nuclei were additionally stained with Hoechst 33342 [21].

### Cell metabolic activity by the MTT assay

Cells were exposed to R6G and R6G-AuNPs for 0.5, 1, 2, 4, and 16 h. Then, the medium was replaced by a 2 mL medium containing 0.5 mg⋅mL^−1^ of 3-(4,5-dimethyl-2-thiazolyl)-2,5-diphenyl-2H-tetrazolium bromide (MTT) followed by incubation cells for 3 h at 37 °C. Afterwards, the medium was carefully removed from the wells. DMSO:ethanol (1:1) was added to each well and the plate was shaked on a plate shaker for 5 min to dissolve formazan crystals. Absorbance of formazan was measured at 560 nm by a Biotec Synergy™ 4 plate reader (www.biotek.com).

### Instrumentation

The electronic absorption spectra of the gold colloid, the R6G solution and the gold colloid mixed with R6G were recorded with a UV–vis-NIR Perkin Elmer spectrophotometer (model Lambda 35) (www.perkinelmer.com) in the range of 190–1,100 nm with a resolution of 2 nm. Quartz cells of 1 cm were used.

Fluorescence images of cells for the quantitative analysis of fluorescence were collected using Olympus Scan^R system (www.olympus-europa.com) in DAPI and Cy3 channels for Hoechst 33342 and rhodamine 6G, respectively. Images were constructed by using Columbus data storage and analysis system (Perkin Elmer). 3D fluorescence images of R6G-AuNPs-incubated cells were acquired with a A1-Si Nikon (Japan) confocal laser scanning system built onto a Nikon inverted microscope Ti-E using a Plan Apo 100×/1.4 Oil DIC objective (www.nikon.com). Images were recorded at a resolution of 1,024 × 1,024 while Hoechst 33342 and R6G were excited with 405 and 561 nm diode lasers, respectively. 3D fluorescence images were constructed by using a NIS-Elements AR 3.2 software.

SERS mapping of cells and SERS spectra of R6G-conjugated AuNPs were carried out by using a WITec system (www.witec.de), equipped with an immersive objective with magnification of 60× and a He-Ne laser (632.8 nm). This optical setting provides max. spatial resolution of 0.4 μm. For all measurements, integration time was 0.05 s with a single accumulation, laser power of 10 mW and grating 600 g⋅mm^−1^. The recorded spectral range for this grating is 0–2,400 cm^−1^. SERS spectra of R6G-AuNPs as a reference for cells mapping were recorded by placing a sample in a glass cuvette and using an air objective (20×). Three SERS spectra were acquired, each for freshly prepared sample. The latter was prepared by mixing 500 μL of the Au colloid with 5 μL of a 1 × 10^−3^ M R6G solution in a glass cuvette. For Raman mapping, raster scans over single living cells were carried out with a computer-controlled *x,y*-stage. The mapping step was 1 μm.

### Analysis of SERS maps

Hierarchical K-means cluster analysis of SERS images was performed by using a WITec Project 2.06 software. The spectra were analysed in the region of 200–1,800 cm^−1^ after a routine procedure for cosmic rays removal and smoothing (13 points) using a Savitzky–Golay algorithm.

## Results and discussion

### Spectral characterisation of R6G-AuNPs

An important step in the synthesis of SERS labels is selection of an appropriate metal surface. The metallic support should be stable, small enough to easily cross the cellular membrane, and cause a relatively low cytotoxicity. In our work gold nanoparticles with the average diameter of 40 nm were chosen. The DLS (Dynamic Light Scattering) method confirmed the size of AuNPs (approx. 45 nm) while AFM images showed clearly the spherical shape of non-aggregated nanoparticles (Fig. S1 in Electronic Supporting Material, ESM). According to DLS, gold nanoparticles exhibited relatively low polydispersity index of 0.38. The UV–vis spectra display the presence of a LSPR band of the colloid at 527 nm, typical for this diameter of the Au sol (Fig. S2, ESM). Then a spectrum of the dye mixed with the gold colloid shows the characteristics of aggregate formation by the presence of extended plasmon band with a second maximum at 641 nm, *c.f.* Fig S2. Formation of small aggregates results in an increase of SERS enhancement factors [22]. Here, the chosen size of AuNPs and concentration of R6G provide a stable SERS signal of the dye. Figure S3 (ESM) shows the comparison of the exemplary three raw SERS spectra of R6G adsorbed on the gold colloid used further in SERS mapping of cells. Positions, full width at half maximum and relative intensities of SERS bands are identical in each spectrum while the total intensity of the spectra in the recorded region varies within *ca.* 7 %. This confirms the stability of the SERS signal of the Raman reporter resulting from a relatively similar aggregation of the gold nanoparticles.

### Cytotoxicity of R6G and R6G-AuNPs

A cytotoxic effect of the gold and silver nanoparticles has been previously reported [23, 24]. The exposition of C17.2 neural progenitor, human umbilical vein endothelial, and PC12 rat pheochromocytoma cells to AuNPs at the concentration of 100 nM for 24 h did not induce acute cytotoxicity as activity of lactate dehydrogenase (LDH) revealed while deformation of actin and tubulin cytoskeleton was observed for 50 nM concentration of AuNPs [24]. However, this effect is time- and concentration-dependent as well as it can be specific for a type of cells. In our work, we used a lower concentration of AuNPs (10 pM in medium) than in the literature [24], therefore we excluded a toxic influence of the gold nanoparticles on the EA.hy926 cells. Next, we evaluated toxicity of R6G and R6G-AuNPs by performing the MTT assay (Table 1). The MTT assay, which is sensitive to mitochondrial activity, exhibits not only cell apoptosis but also the inhibition of cell division. The EA.hy926 cells are sensitive to rhodamine 6G after long incubation time whereas the presence of both the dye and the gold nanoparticles affects the cells even after 1-h contact. Non-toxic effect of the SERS label is observed up to 4 h and then massive apoptosis/necrosis of the endothelial cells takes place after 16 h of incubation. This indicates that EA.hy926 cells can be exposed to nanocarries in a relatively short time of a few hours.

**Table 1:** Cell viability determined by a MTT assay for cells incubated with R6G-AuNPs and R6G in time intervals. Control cells were incubated with a pure medium

Incubation time [h]	Concentration of formazan [% of control]	
	R6G-AuNPs	R6G
0.5	97	100
1	79	100
2	73	90
4	77	90
16	37	83
Control	100	100

### The uptake of R6G and R6G-AuNPs by endothelial cells: 2D quantitative intracellular fluorescence

**Fig. 1:**
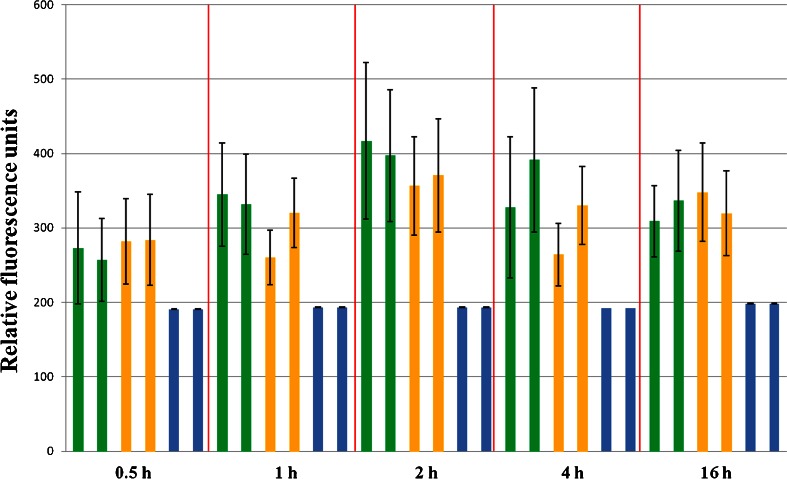
The comparison of R6G-AuNPs (*green*) and R6G (*yellow*) uptake by EA.hy 926 endothelial cells for 0.5–16 h in 37 °C. Control cells (*blue*) were incubated in a label-free medium. The relative fluorescence intensities are provided along with standard deviation bars

A crucial step in the application of the dyes and nanoparticles in detection of intracellular chemical environment is determination of the uptake mechanism of non-conjugated and conjugated dye molecules to Au nanoparticles. For this purpose 2D quantitative fluorescence spectroscopy can be used to determine an amount of a labile fluorescent reporter inside cells. However, such information cannot be achieved for the bare metal nanoparticles as they do not possess the fluorescent features. In our approach, we employed incubation time from 0.5 till 16 h (0.5, 1, 2, 4 and 16 h) keeping cells in an optimal temperature of 37 °C as well as under energy depletion conditions at 25 and 4 °C. This experiment should show: 1/ what time is required to saturate the endothelial cells by the dye, 2/ differences in kinetics of the uptake, consequently in the membrane transport, between conjugated and non-conjugated molecules of the dye, and finally 3/ the contribution of the active (involving cellular energy) and passive (a diffusive process) mechanism into the membrane transport of the endothelium. An overall summary of the kinetics of the uptake of non-conjugated and conjugated R6G molecules for the EA.hy926 cells is given in Fig. 1. During uptake of the dye, the fluorescence rises quite quickly on the timescale of 30 min in comparison to the control group indicating the presence of rhodamine 6G in the intracellular compartments of endothelial cells, and then the intensity of fluorescence emission for R6G continues to grow up to 2 h. With a longer than 2 h incubation time, it is maintained at a relatively similar level indicating that saturation of the cells by the dye takes place within 2 h. In terms of the export of the Raman reporter from the endothelial cells, a very slow decay process can occur after 16 h as an insignificant fluorescence decrease is found for this incubation time. Moreover, the fluorescence emission is very similar for both R6G and R6G-AuNPs on the whole timescale used in our protocol. This undoubtedly confirms the intracellular transport of a similar amount of the dye regardless of its binding to the gold nanoparticles in the medium. However, these data do not indicate whether the R6G-AuNPs or the bare R6G molecules are transported through the cellular membrane since the technique is not sensitive to the metallic SERS support. This will be confirmed by SERS mapping in the section below.

To assess what mechanism, i.e. cellular chemical–mechanical energy or a simple diffusion process, plays a dominant role in the uptake of the dye/nanoparticles, the fluorescence was measured for cell cultures at 25 and 4 °C (Fig. S4, ESM). Unfortunately, endothelial cells are very sensitive to the temperature stress and after 1 h the cellular necrosis occurred at room temperature. For cells incubated at 4 °C we were able to carry out further measurements even after 4 h. At 4 °C all the cellular processes are slower, so the necrosis process appears later comparing to 25 °C.

The average intensity of fluorescence emission for cells incubated with R6G-AuNPs as well as with R6G for 30 min at 37, 25 and 4 °C is 265/283, 217/203 and 221/221 [in relative fluorescence intensity], respectively for R6G-AuNPs/R6G, while the control group exhibits autofluorescence with the intensity of 193 regardless of temperature (Fig. 1 and Fig. S4, ESM). These results indicate that no clear trend can be found between the uptake of R6G and R6G-AuNPs at a given temperature in short time of the incubation. The fluorescence only slightly decreases along with inducing energy depletion. After the 1 h incubation, a higher concentration of R6G conjugated to the gold nanoparticles than the bare R6G is found in the endothelial cells incubated at 37 and 25 °C since relative fluorescence intensities are 338/290 and 260/238, respectively. The results also suggest that a principal mechanism of cellular uptake of R6G-AuNPs but not of R6G is an energy-dependent process of endocytosis. It is well known that the mechanism of endocytosis depends on the size of material and for NPs with a diameter smaller than 40 nm, the docking of the NPs does not produce enough energy to completely wrap the NPs by the cell membrane [26, 27]. In our work, the gold nanoparticles are of a *ca*. 40 nm diameter and they aggregate after mixing with the Raman reporter. Moreover, a very slight increase in fluorescence in comparison to the control group is observed for the cells incubated at 4 °C (212/226 for R6G-AuNPs/R6G). In this case, however, the fluorescence intensity has no obvious change compared to that observed for the 0.5-h incubation but it increases after 2 h, *c.f.* Fig. S4B (ESM), indicating a slow diffusion process. At the present stage, it is very hard to clarify whether the difference in the fluorescence results between the cells with R6G-AuNPs and R6G is statistically significant (approx. 250–300 cells measured per well). The cellular delivery of R6G-AuNPs to the endothelium via active and passive mechanisms may occur simultaneously in our experiment leading to the saturation of the cells after 2–4 h.

**Fig. 2:**
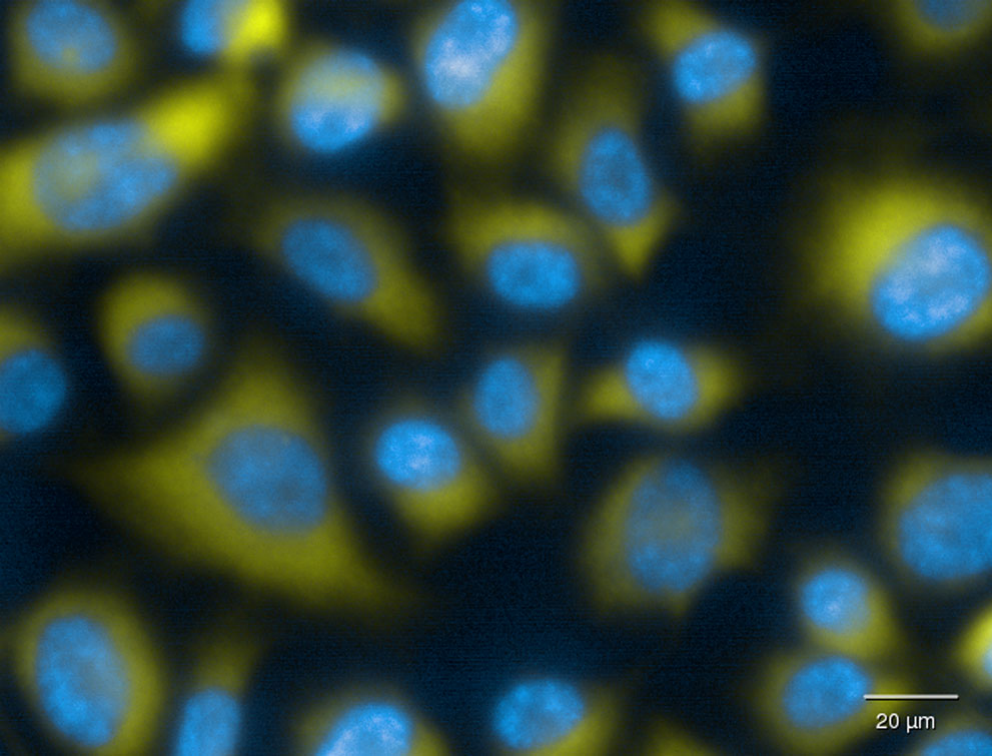
The rhodamine 6G molecules detected in cytoplasm of EA.hy 926 endothelial cells by 2D fluorescence microscopy. Nuclei stained with Hoechst 33342 are blue, R6G is yellow

The 2D intracellular fluorescence measurements of cells with the use of a high-content screening automated microscope enable the quantitative analysis of a dye for a large cell population [28, 29], however, this technique does not determine an exact location of the dye molecules in the intracellular compartment. Figure 2 illustrates fluorescence image of the EA.hy 926 cells with the Hoechst-stained nuclei and yellow staining of R6G distributed within cytoplasm. It is rather impossible to asses from this image whether the R6G molecules are present inside the cells, or possibly they are simply attached to the membrane surface of the endothelial cells.

### The location of R6G in the endothelial cells: 3D confocal fluorescence

**Fig. 3:**
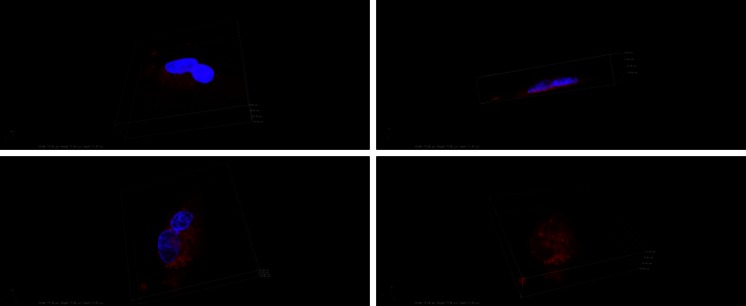
3D confocal fluorescence images of endothelial cells incubated for 2 h with R6G-AuNPs. *Blue*: nuclei stained with Hoechst, *red*: rhodamine 6G. The right bottom image shows the distribution of R6G only

3D confocal microscopy was employed to confirm the intracellular uptake of R6G-AuNPs by the endothelial cells. Figure 3 illustrates location of the nucleus surrounded by the dye spread throughout the cell without entering to the nucleus. The changes in the fluorescence intensity show that small aggregates of the Raman reporter dye are distributed unevenly in the cytoplasm but it is rather impossible to determine if they are localised in specific cell compartments.

### Chemical information: SERS mapping of the endothelial cells

**Fig. 4:**
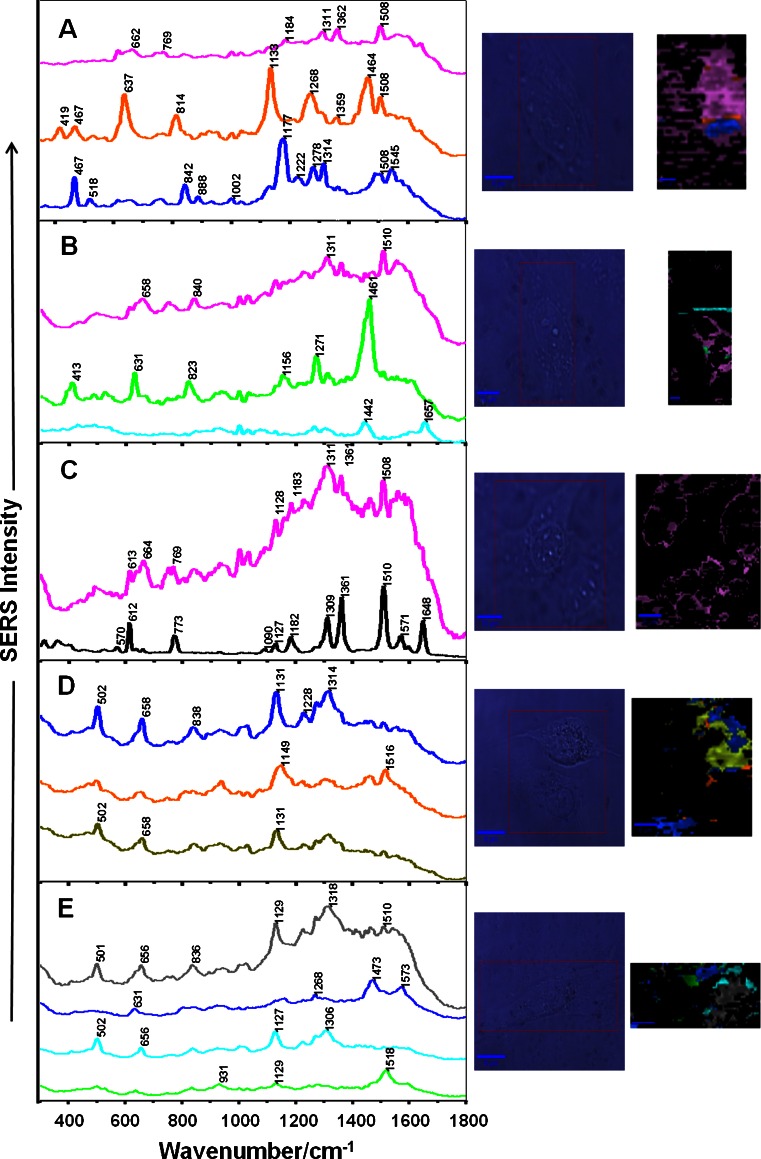
SERS mean spectra extracted from cluster maps of endothelial cells incubated with R6G-AuNPs for 0.5 h (**a**), 1 h (**b**), 2 h (**c**), 4 h (**d**), and 16 h (**e**) along with a microphotography of a cell and a cluster map. Black trace in (**c**) represents a reference SERS spectrum of rhodamine 6G. The colours of spectra correspond to the colours of classes in the cluster map

As shown above, the fluorescence provides information on the cellular distribution of rhodamine 6G but this technique does not differentiate the bare and conjugated dye so it is difficult to follow the presence/absence of R6G conjugated to AuNPs. To investigate this as well as to analyse potential chemical information gathered from molecular vibrations of biomolecules, surface-enhanced Raman mapping was performed for the endothelial cells incubated with R6G-AuNPs. The control groups were the cell cultures incubated in the label-free medium as well as in the medium containing R6G. For both groups, no Raman signal was recorded indicating that the use of the 633 nm laser excitation and short integration time (0.05 s) do not provide intracellular chemical information without a SERS support (data not shown). This has been also reported by Sathuluri et al. [30]. Figure 4 illustrates microscopic images of representative cells along with K-means cluster maps and mean SERS spectra extracted from the corresponding classes. The SERS bands of rhodamine 6G are observed in the spectra recorded for the incubation time up to 2 h. Their distributions inside the cells shown in the cluster maps implicates a decrease in the concentration of the R6G-AuNPs particles upon long incubation time and moving them towards the cell membrane, *cf.* pink traces in Fig. 4a–c. On the other hand, since the SERS spectra for each incubation time exhibit the presence of bands attributed to various biomolecules (discussed below), the R6G molecules must be de-attached from the surface of the gold and then biomolecules present in the close vicinity of hot spots exhibit SERS signal. In our preliminary studies on probing cellular environment of the endothelium by the R6G-AuNPs label, we observed the SERS signal of the dye for the 2- and 6-h incubation time while SERS bands originating from various biomolecules were observed up to 16 h [8]. This indicates that the conjugation of rhodamine 6G to nanoparticles can be stable up to a few hours. Salvati et al. have shown that after the uptake of nanoparticles containing a high concentration of a labile dye, the dye molecules are randomly spread across the intracellular space whereas the dye conjugated to nanoparticles is spatially localised [25]. A similar effect is observed in our experiment. As the 3D fluorescence image showed above, the labile R6G molecules fill up the cytoplasm, probably reaching the endosomes of the cell whereas the distribution of R6G-AuNPs in SERS maps is localised.

Apart from SERS bands of R6G, mean SERS spectra show the presence of marker bands of cell biomolecules assigned according to the literature, *see* Fig. 4 [8, 9, 29, 30]. In the spectra of the cells incubated for 30 min., bands attributed to proteins and lipids (1,268, 1,278, 1,464, 1,545 cm^−1^) and the phosphate groups (419, 814 cm^−1^) are found (Fig. 4a). After the 1-h incubation, protein and lipid bands are still observed (at 1,271, 1,442, 1,461, 1,657 cm^−1^) along with Raman markers for tyrosine (631, 823 cm^−1^) and the C-C and C-N stretching vibrations (1,156 cm^−1^), *c.f.* Fig. 4b. In turn, a few bands originating from Cys (664 cm^−1^), Trp (760 cm^−1^) and Phe (1,001 and 1,021 cm^−1^) appear in SERS spectrum of the cells upon the 2-h incubation with R6G-AuNPs but this spectrum is mainly dominated by the R6G signal (Fig. 4c). For the 4 and 16-h incubation (Fig. 4d, e), we identified bands of amino acid residues such as cysteine (502 cm^−1^), tyrosine (658, 838 cm^−1^), proline (931 and ca. 1,130 cm^−1^) and purines/pyridines (1,228, 1,268, 1,314, 1,516 cm^−1^). Summarizing, the SERS label containing R6G as a Raman reporter enables sensitive and locally confined chemical probing of intracellular environment indicating mainly the presence of amino acid residues and moieties of nucleic acids.

## Conclusion

The work demonstrates a detailed and multiparametric approach to assess the interactions of the endothelial cells with the gold nanoparticles immobilized with the Raman reporter—rhodamine 6G. Firstly, we show that the designed particles can interfere with normal cellular homeostasis within 4 h, leading to substantial toxic effect after 16 h. This suggests that although a non-toxic concentration of AuNPs is used in the experiment, their conjugation with a Raman reporter can induce cytotoxic mechanism in the endothelium especially during the long-time incubation, further emphasizing the importance of evaluating the toxic effects of nanomaterials on cultured cells. Then, we employ the chosen nanoparticles to study the cellular uptake behaviour of R6G-AuNPs by means of 2D and 3D fluorescence and SERS spectroscopy. We reveal that the uptake of the dye as well as the dye—Au support is a relatively fast process in that the cell is saturated by both species within 2 h and they are accumulated in the cytoplasm without an indication of a specific cellular compartment. The current work suggests that the mechanism of the cellular uptake is energy-dependent endocytosis. However, specific pathways of such a mechanism must be studied separately. In turn, intracellular SERS imaging provides the strong and stable signal of cellular components of the endothelium exhibiting the presence of amino acid residues and bases of nucleic acids. The comparison of both SERS and fluorescence imaging clearly shows that the Raman reporter is de-attached from the surface of SERS substrate in the intracellular environment already after 0.5 h of the incubation since SERS signal of biomolecules is observed. This may implicate a real-time process of exchanging adsorbates in the local optical field of the metal nanoaggregates and/or rearrangement of gold nanoaggregates in a way in which SERS hot spots are formed around other molecules than the dye. In summary, our studies also demonstrate the strength of a multi-methodological approach for in vivo cell labelling applications and may provide hints to establish a reference protocol for further studies investigating interactions between cells and nanocarries.

## Electronic supplementary material

Below is the link to the electronic supplementary material.

ESM 1 (PDF 552 kb)

## References

[1] Auchinvole CAR, Richardson P, McGuinnes C, Mallikarjun V, Donaldson K, McNab H, Campbell CJ (2012). Monitoring intracellular redox potential changes using SERS nanosensors. ACS Nano.

[2] Leopold N, Baena JR, Bolboaca M, Cozar O, Kiefer W, Lendl B (2004). Raman, IR, and surface-enhanced Raman spectroscopy of papaverine. An automated setup for in situ synthesis of the silver substrate and recording of the SER spectra. Vib Spectrosc.

[3] Culha M, Cullum B, Lavrik N, Klutse CK (2012). Surface-enhanced Raman scattering as an emerging characterization and detection technique. J Nanotechnol.

[4] Jaworska A, Malek K, Marzec KM, Baranska M (2012). Nicotinamide and trigonelline studied with surface-enhanced FT-Raman spectroscopy. Vib Spectrosc.

[5] Kneipp J, Kneipp H, Kneipp K (2008). SERS—a single-molecule and nanoscale tool for bioanalytics. Chem Soc Rev.

[6] Guo X, Guo Z, Jin Y, Liu Z, Zhang W, Huang D (2012). Silver–gold core-shell nanoparticles containing methylene blue as SERS labels for probing and imaging of live cells. Microchim Acta.

[7] Kneipp K, Haka AS, Kneipp H, Badizadegan K, Yoshizawa N, Boone C, Shafer-Peltier KE, Motz JT, Dasari RR, Feld MS (2002). Surface-enhanced Raman spectroscopy in single living cells using gold nanoparticles. Appl Spectrosc.

[8] Jaworska A, Malek K, Kachamakova-Trojanowska N, Chlopicki S, Baranska M (2013). The uptake of gold nanoparticles by endothelial cells studied by surface-enhanced Raman spectroscopy. Biomed Spectrosc Imaging.

[9] Malek K, Jaworska A, Krala P, Kachamakova-Trojanowska N, Baranska M (2013). Imaging of macrophages by Surface Enhanced Raman Spectroscopy (SERS). Biomed Spectrosc Imaging.

[10] Schlucker S (2011). Surface enhanced Raman spectroscopy.

[11] Kneipp J, Kneipp H, Rajadurai A, Redmond RW, Kneipp K (2009). Optical probing and imaging of live cells using SERS labels. J Raman Spectrosc.

[12] Halbhuber KJ, Konig K (2003). Modern laser scanning microscopy in biology, biotechnology and medicine. Ann Anat.

[13] Pygall SR, Whetstone J, Timmins P, Melia CD (2007). Pharmaceutical applications of confocal laser scanning microscopy: the physical characterisation of pharmaceutical systems. Adv Drug Deliv Rev.

[14] Fischer RS, Wu Y, Kanchanawong P, Shroff H, Waterman CM (2011). Microscopy in 3D: a biologist’s toolbox. Trends Cell Biol.

[15] Conchello JA, Lichtman JW (2005). Optical sectioning microscopy. Nat Methods.

[16] Foldes-Papp Z, Demel U, Tilz GP (2003). Laser scanning confocal fluorescence microscopy: an overview. Int Immunopharmacol.

[17] Félétou M (2011) Multiple functions of the endothelial cells. In: The endothelium part 1: multiple functions of the endothelial cells—focus on endothelium-derived vasoactive mediators. Morgan & Claypool Life Sciences, San Rafael 21850763

[18] Wnuczko K, Szczepanski M (2007). Endothelium—characteristics and functions. Pol Merk Lek.

[19] Frens G (1973). Controlled nucleation for the regulation of the particle size in monodisperse gold suspensions. Nat Phys Sci.

[20] Edgell C, McDonald C, Graham J (1983). Permanent cell line expressing human factor VIII-related antigen established by hybridization. Proc Natl Acad Sci.

[21] Otto F, Tsou KC (1985). A comparative study of DAPI, DIPI, and Hoechst 22358 and 33342 as chromosomal DNA stains. Stain Technol.

[22] Joseph V, Matschulat A, Polte J, Rolf S, Emmenrling F, Kneipp J (2011). SERS enhancement of gold nanospheres of defined size. J Raman Spectrosc.

[23] Asare N, Instanes C, Sandberg WJ, Refsnes M, Schwarze P, Kruszewski M, Brunborg G (2012). Cytotoxic and genotoxic effects of silver nanoparticles in testicular cells. Toxicology.

[24] Soenen SJ, Manshian B, Montenegro JM, Amin F, Meermann B, Thiron T, Cornelissen M, Vanhaecke F, Doak S, Parak WJ, De Smedt S, Braeckmans K (2012). Cytotoxic effects of gold nanoparticles: a multiparametric study. ACS Nano.

[25] Salvati A, Aberg C, dos Santos T, Varela J, Pinto P, Lynch I, Dawson KA (2011). Experimental and theoretical comparison of intracellular import of polymeric nanoparticles and small molecules: toward models of uptake kinetics. Nanomedicine.

[26] Wang SH, Lee Ch W, Chiou A, Wei PK (2010). Size-dependent endocytosis of gold nanoparticles studied by three-dimentional mapping of plasmonic scattering images. J Nanobiotechnol.

[27] Huang J, Zong C, Shen H, Liu M, Chen B, Ren B, Zhang Z (2012). Mechanism of cellular uptake of grapheme oxide studied by surface-enhanced Raman spectroscopy. Small.

[28] Xu Z, Huang X, Dong C, Ren J (2014). Fluorescence correlation spectroscopy of gold nanoparticles, and its application to an aptamer-based homogeneous thrombin assay. Microchim Acta.

[29] Kneipp J, Kneipp H, McLaughlin M, Brown D, Kneipp K (2006). In vivo molecular probing of cellular compartments with gold nanoparticles and nanoaggregates. Nano Lett.

[30] Sathuluri RR, Yoshikawa H, Shimizu E, Saito M, Tamiya E (2011). Gold nanoparticle-based surface-enhanced Raman scattering for noninvasive molecular probing of embryonic stem cell differentiation. PLoS One.

