# Supplementary material for: Rhodamine 6G conjugated to gold nanoparticles as labels for both SERS and fluorescence studies on live endothelial cells
Source: Mikrochim Acta. 2014 Jun 19;182(1):119–27. doi: 10.1007/s00604-014-1307-5 (PMC4281367; doi:10.1007/s00604-014-1307-5)
Supplement: Supplementary file 1 — (PDF 552 kb) [file 604_2014_1307_MOESM1_ESM.pdf]

Electronic Supporting Material

Rhodamine 6G conjugated to gold nanoparticles as labels for both SERS and fluorescence studies on live endothelial cells

Aleksandra Jaworska[1,2], Tomasz Wojcik[1], Kamilla Malek[1,2]*, , Urszula Kwolek[2], Mariusz Kepczynski[1,2], Abu Ayoobul Ansary[2], Stefan Chlopicki[1,3], Malgorzata Baranska[1,2]

[1] *Jagiellonian Centre for Experimental Therapeutics, Jagiellonian University, Bobrzynskiego 14, 30-348 Krakow, Poland*

[2] *Faculty of Chemistry, Jagiellonian University, Ingardena 3, 30-060 Krakow, Poland*

[3] *Department of Experimental Pharmacology, Jagiellonian University Medical College, Grzegorzecka 16, 31-531 Krakow, Poland*

*Author to whom the correspondence should be sent: malek@chemia.uj.edu.pl

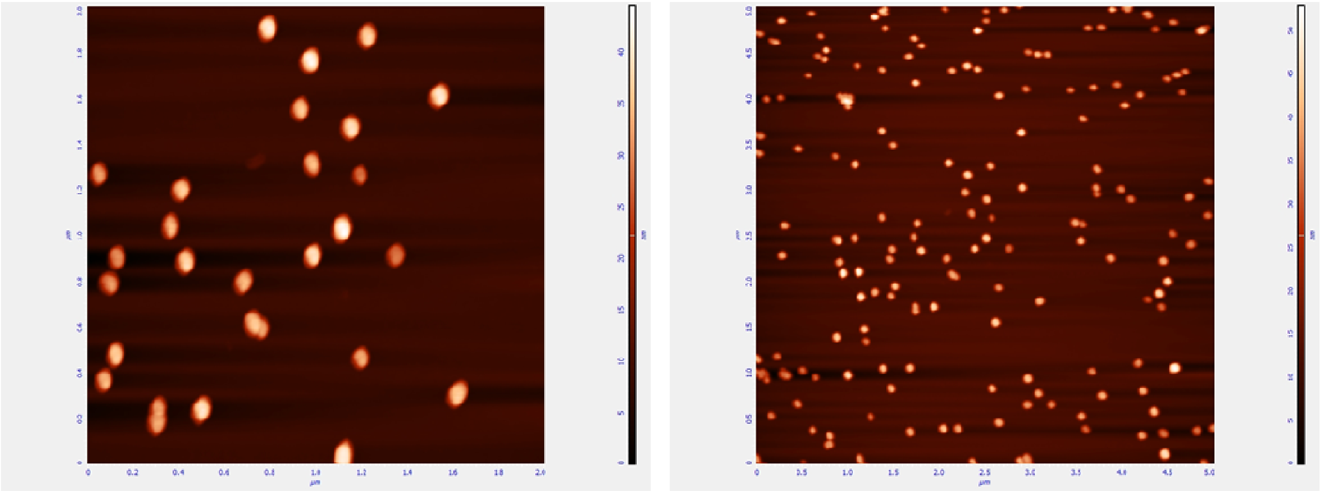

Figure S1. AFM images of gold nanoparticles.

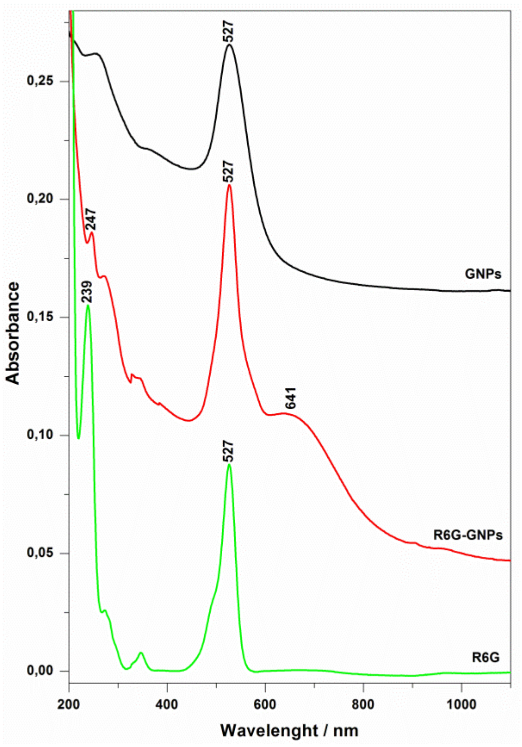

Figure S2. UV-Vis spectra of the gold colloid (GNPs), gold nanoparticles conjugated to rhodamine 6G (GNPs-R6G) and rhodamine 6G (R6G).

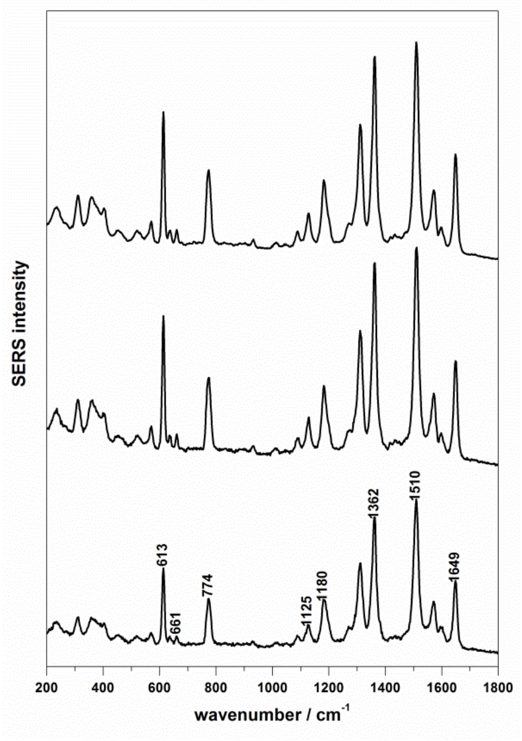

Figure S3. Raw SERS spectra of rhodamine 6G adsorbed on the Au colloid recorded for three samples.

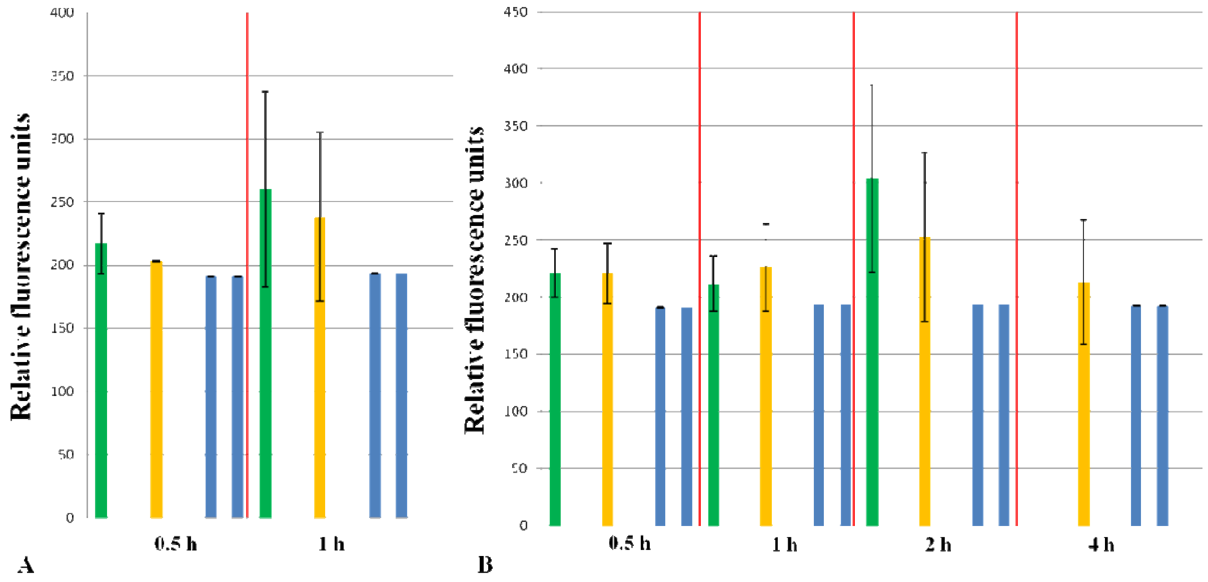

Figure S4. The comparison of R6G-GNPs (green) and R6G (yellow) uptake by EA.hy 926 endothelial cells for 0.5 and 1 h in 25 °C (**A**) and for 0.5 - 4 h in 4 °C (**B**). Control cells (blue) were incubated in a label-free medium. The relative fluorescence intensities are provided along with standard deviation bars.
